# Mismatch repair genes status in sporadic Saudi colorectal cancer patients

**DOI:** 10.1186/1471-2164-15-S2-P57

**Published:** 2014-04-02

**Authors:** Manar Ata, Ashraf Dallol, Jaudah Al-Maghrabi, Abdulrahmn Al-Sibiany, Mahmoud Al-Ahwal, Mohammed Al-Qahtani, Abdelbaset Buhmeida

**Affiliations:** 1 Center of Excellence in Genomic Medicine Research, King Abdulaziz University, Jeddah, Saudi Arabia; 2 Department of Pathology, King Abdulaziz University, Jeddah, Saudi Arabia; 3 Department of Surgery, King Abdulaziz University, Jeddah, Saudi Arabia; 4 Department of Oncology, King Abdulaziz University, Jeddah, Saudi Arabia

## Background

Colorectal cancer (CRC) is becoming a major public health problem globally [1]. It ranks first in men and third in women of Saudi population according to Saudi Cancer Registry 2009 [2]. Many efforts have been directed toward assessment of best strategies for establishing molecular prognostic model [3,4]. In our study, we aimed to assess the prevalence of Mismatch Repair (MMR) genes defects in CRC and evaluate the concordance rate between Microsatellite Instability (MSI) and Immunohistochemistry (IHC).

## Materials and methods

Two techniques were used: IHC using antibodies for MLH1 and MSH2 gene proteins and MSI tests using a panel of five Microsatellites' markers (BAT25, BAT26, D5S346, D2S123 and D17S250) that have been validated and recommended for tumor characterization as MS-stable, MSI-H or MSI-L. DNA samples from tumors and normal tissues of 83 patients undergoing curative surgery of sporadic CRC were amplified using Polymerase chain reaction (PCR). PCR products were run on ABI prism 310 sequencer and GeneScan; version 310 software was used for analysis. Paraffinized tissue sections slides from the same patients tissues were used for IHC detection of MLH1 and MSH2 proteins.

## Results

The prevalence of MSI-H, MSI-L and MS-stable were 44.6 %, 31.3 % and 24.1 % respectively. IHC identified 50 cases (60.2%) with loss of expression of MLH1 and 40 cases (48.2%) with loss of expression of MSH2. Concordance rate between MSI and IHC is 55.4%. Furthermore, 28.9% of no concordance is attributed to other MMR genes not included in our study.

## Conclusions

MSI and IHC are complementary useful techniques for evaluation of MMR status. Further study with a large cohort is needed to evaluate correlation with clinicopathological characteristics and to be used as prognostics.

*This work was financially supported by King Abdulaziz City for Science and Technology* (*KACST*) *under research no* (*ARP -30-262*)*.*
